# Calibration Methods for Low-Cost Particulate Matter Sensors Considering Seasonal Variability

**DOI:** 10.3390/s24103023

**Published:** 2024-05-10

**Authors:** Jiwoo Kang, Kanghyeok Choi

**Affiliations:** Department of Geoinformatic Engineering, Inha University, 100 Inha-ro, Michuhol-gu, Incheon 22212, Republic of Korea; bum3201@inha.edu

**Keywords:** particulate matter, low-cost sensor, calibration, seasonal variability, environment factors

## Abstract

Many countries use low-cost sensors for high-resolution monitoring of particulate matter (PM_2.5_ and PM_10_) to manage public health. To enhance the accuracy of low-cost sensors, studies have been conducted to calibrate them considering environmental variables. Previous studies have considered various variables to calibrate seasonal variations in the PM concentration but have limitations in properly accounting for seasonal variability. This study considered the meridian altitude to account for seasonal variations in the PM concentration. In the PM_10_ calibration, we considered the calibrated PM_2.5_ as a subset of PM_10_. To validate the proposed methodology, we used the feedforward neural network, support vector machine, generalized additive model, and stepwise linear regression algorithms to analyze the results for different combinations of input variables. The inclusion of the meridian altitude enhanced the accuracy and explanatory power of the calibration model. For PM_2.5_, the combination of relative humidity, temperature, and meridian altitude yielded the best performance, with an average R^2^ of 0.93 and root mean square error of 5.6 µg/m^3^. For PM_10_, the average mean absolute percentage error decreased from 27.41% to 18.55% when considering the meridian altitude and further decreased to 15.35% when calibrated PM_2.5_ was added.

## 1. Introduction

Particulate matter (PM) is a primary factor that influences air quality and has a significant impact on public health [1,2,3]. Many countries have recognized the need for PM monitoring systems and built sophisticated observation infrastructures to support PM management and mitigation [4,5]. However, the installation and maintenance of high-precision PM sensors is costly, and the large size of the sensors limits their installation locations [5,6]. This limits the spatial resolution of the PM concentration monitoring. In Korea, the average area of precision sensors installed at national observatories is only 11 km², which makes it challenging to provide PM information that affects the public [7].

Recently, low-cost sensors (LCSs) have been increasingly utilized as alternatives for high-resolution PM monitoring [8,9]. LCSs are economical and small and can be densely deployed, allowing the observation of PM with high spatial resolution [9,10]. However, the PM LCS is relatively inaccurate compared with national observatories [8,9,11]. Calibration methodologies have been proposed to improve the accuracy of the PM LCS. The approaches of existing studies can be classified into the optimization of input variables and proposal of calibration models.

The first approach to improve the calibration performance is to select and combine different environmental variables as input variables. The PM LCS uses light scattering to measure the size and concentration of the PM. The accuracy of these observations was influenced by various environmental variables [9,12,13,14]. Relative humidity (RH) is a crucial environmental factor commonly considered in PM LCS calibrations. This significantly affects the accuracy of observations by altering the size and refractive index of hygroscopic particles [15,16,17,18]. Therefore, most studies on LCS calibration have selected RH as the input variable [19]. In addition, previous studies have proposed calibration methodologies that use RH and T as input variables [20,21,22,23,24]. Several studies have attempted to improve calibration accuracy by incorporating wind speed and wind direction in addition to RH and T [4,25,26,27,28].

Calibration methods can be categorized into physical-mechanism-based and statistical models [29]. Physical-mechanism-based models are typically calibrated using a correction factor to compensate for the bias caused by RH. Methodologies that use correction factors require relatively low computational power and are easy to apply; therefore, they have been used in several studies [15,30,31,32,33,34,35,36]. Crilley et al. and Venkatraman et al. proposed RH correction factor-based calibration using the κ -Köhler theory [31,34,35]. In addition, Laulainen et al. and Chakrabarti et al. performed PM concentration calibrations using empirical RH correction factors [36,37]. These methodologies have the advantage of being simple and efficient, requiring only RH information; however, they are generally less accurate than other methodologies [10].

Statistical models include linear regression (LR) and machine learning and typically utilize environmental factors, such as RH. These models can generally achieve higher accuracy than correction factor methods and have been used in several studies. Several studies have conducted PM calibrations using LR and multiple LR (MLR) [20,38,39]. Liu et al. used stepwise LR (SLR) to suggest appropriate combinations of input variables for each pollutant, considering pollutant concentrations and meteorological factors, and established calibration models for different types of pollutants [40]. Jiang et al. used SLR with RH and T to improve the accuracy of LCS calibration [41]. Badura et al. used SLR to select the optimal combination of input variables, enabling them to select a model that balances brevity and precision [42]. Chen et al. considered the effects of RH, T, wind speed, and wind direction on PM concentrations and used LR, a support vector machine (SVM), and a feedforward neural network (FNN), with the FNN having the highest accuracy [25]. Mahajan et al. compared LR, an artificial neural network (ANN), support vector regression (SVR), and random forest (RF) to select a suitable calibration model for PM_2.5_ and found that SVR performed the best [43]. Munir et al. used LR and a generalized additive model (GAM) with meteorological variables to calibrate air quality sensors; the GAM effectively improved the accuracy of the sensors [27].

Several studies have contributed to the effective calibration of a PM LCS by developing calibration models, conducting comparative analyses using different methodologies, and considering important environmental factors. However, several challenges remain to be solved in order to further improve accuracy and efficiency. One of the challenges is the seasonal variability of PM concentrations, which significantly affects calibration accuracy [44,45]. The seasonal variation of PM concentrations is especially striking in regions with distinct climate variations. For example, in South Korea, PM concentrations and composition significantly change seasonally due to the arrival of fine dust from neighboring countries in spring, the rainy season in summer, and heating activities in winter [46]. Previous studies have considered seasonal variation to account for PM measurements, but this variability is not accounted for in the PM calibration [27,38,47]. Meanwhile, some studies have considered seasonal variations in calibration, but they have the following limitations. Kumar et al. have developed seasonal calibration models by selecting meteorological variables that have a significant impact on each season [43]. Also, Srishti et al. converted each season into a dummy variable and included it as an input variable in the calibration model [48]. However, these approaches have limitations in properly accounting for the continuous and gradually changing seasonal variability, as the seasons are categorized based on a specific point in time. In another study, Considine et al. converted the month, week, and hour variables into a periodic function such as sine or cosine to account for continuous temporal variation [49]. But the periodic variables introduced in this study did not contribute to the improvement in calibration accuracy, except when considering the additional regional characteristics of LCS installation.

This study aimed to improve the accuracy and efficiency of PM LCS calibration. To achieve this goal, this study proposes a calibration methodology that can effectively account for seasonal variations in the PM concentration using the following approach: First, this study introduced meridian altitude (MA) as an input variable to consider seasonal variation in the calibration. Second, the calibrated PM_2.5_ concentration was used as an additional input variable in the PM_10_ calibration to enhance performance. We analyzed the results by applying these methodologies to representative calibration models and verified the effectiveness of the proposed methodologies.

## 2. Input Variables of Calibration

### 2.1. PM Observation and Reference Data

**Figure 1:**
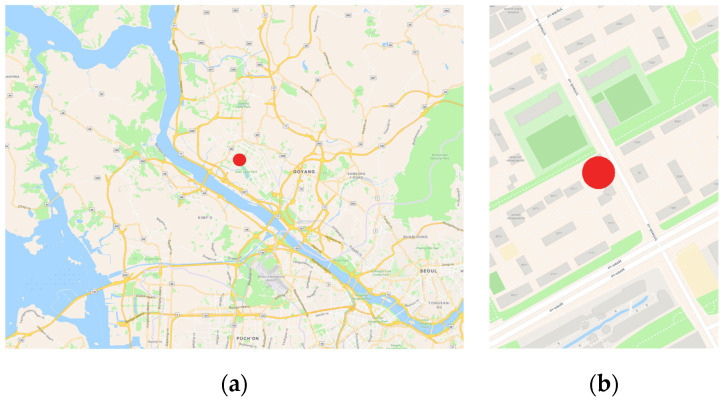
Map of study area: (**a**) map of location and (**b**) map of localization showing site where sensors were installed, marked by red circle (map was developed in ArcGIS Online).

**Figure 2:**
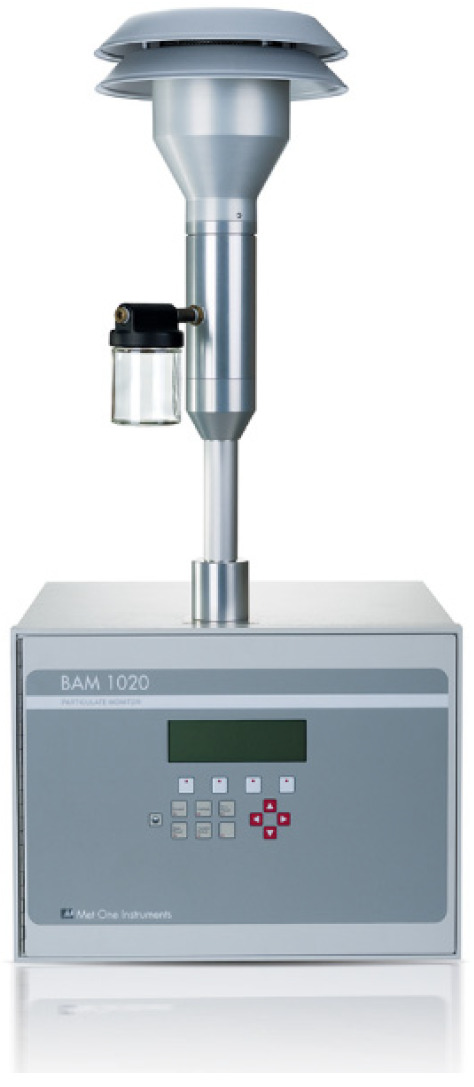
The reference PM sensor (BAM-1020 of Met One Instruments). Here is the manufacturer website: https://metone.com/products/bam-1020/ (accessed on 9 May 2024).

PM_2.5_ and PM_10_ observations and a reference dataset collected over two years (January 2021 to December 2022) were used to train and validate the calibration model. Observations from January 2021 to December 2021 were used for training data, and observations from January 2022 to December 2022 were used for validation data. Both the observation and reference data were acquired from Il-San, South Korea, with each data acquisition being approximately 30 m apart from the other (Figure 1). The reference data were obtained from the BAM-1020 of Met One Instruments (Figure 2). BAM-1020 is a U.S. Environmental Protection Agency (EPA)-approved Federal Equivalent Method (FEM) instrument that is installed and maintained at a national observation station in Korea [4]. The BAM-1020 provides hourly measurements and has a minimum detection limit of 4.8 µg/m^3^. The Korea Meteorological Administration conducts a process to select and validate the measurements from the BAM-1020. This process converts negative observations to zero and retains positive observations that are below the detection limit. In this study, the processed observations were used.

**Figure 3:**
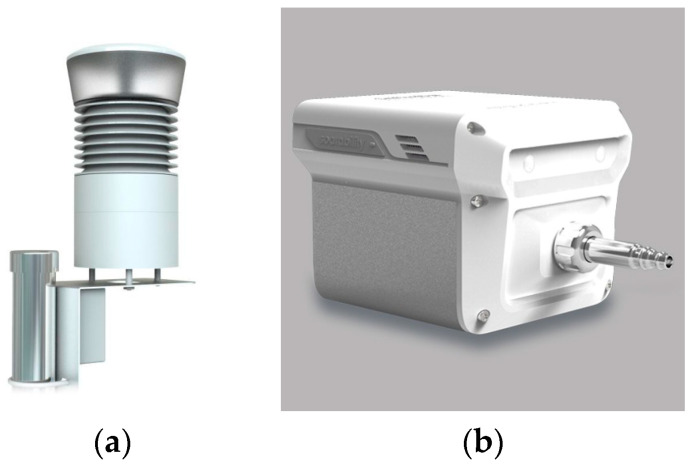
PM LCS used in this study: (**a**) Air-ruler AM100 and (**b**) Sniffer 4D. Here are manufacturer websites for each sensor: http://www.iplug.com/mat/iplug120612/product_03.php (accessed on 9 May 2024) (AM100) and https://sniffer4d.eu/sniffer4d/ (accessed on 9 May 2024) (Sniffer4D).

This study validates the proposed methodology using the Air-Ruler AM100 and Sniffer4D (Figure 3). Both sensors use light scattering to measure PM concentrations, operating within the range of 0.3–1000 µg/m^3^ with a minimum detection limit of 1 µg/m^3^. Each sensor and its observations were characterized (Table 1). AM100 provides PM_10_ and PM_2.5_ measurements, along with ambient humidity and T, at 1 min intervals. The AM100 sensor was observed full-time for two years, except for 1–3 h of maintenance time per week. The Sniffer 4D sensor provides PM, T, and humidity at 1 s intervals. To align the temporal resolution of the two sensor observations, the Sniffer 4D observations were averaged per minute. Sniffer 4D observations were performed at a frequency of approximately 2–3 days per week for the same period as AM100.

Meanwhile, both sensors have been evaluated by an institution or laboratory. The AM100 sensor was evaluated for accuracy by the Korea Environment Corporation, resulting in a rating of 90.1% (Incheon, Korea). The Sniffer4D sensor was assessed against the Thermo Scientific Super Station by Jinan University in China over a period of approximately 180 days (Guangzhou, China). The results showed R^2^ values of 0.95 and 0.88 for PM_2.5_ and PM_10_, respectively [50]. Statistics on the mean, standard deviation, standard error, and measurements below the detection limit for PM_2.5_ and PM_10_ observations using BAM-1020, AM100, and Sniffer4D sensors can be found in Table 2.

### 2.2. Selection of Calibration Method

Various calibration methods were compared through pre-evaluation, and based on the results, the methods were selected for the main experiment. The pre-evaluation includes two methods based on physical mechanisms and four statistical methods. For the physical mechanisms, the approaches of Crilley et al. [31] and Laulainen et al. [36] using RH correction factors were adopted. For the statistical methodologies, the FNN [25], SVR [43], GAM [26], and SLR [40] algorithms used in the respective references were selected. The methods mentioned above were calibrated by considering RH as the only input variable. The performance of each method was assessed using the root mean square error (RMSE), mean absolute percentage error (MAPE), and R-squared (R^2^). This study used MATLAB R2024a software to implement and evaluate the algorithms. Each metric is as shown in Equations (1)–(3).

(1) $$\mathrm{RMSE}=\sqrt{\frac{1}{m}\sum_{i=1}^{m}{\left(y_{i}-{\hat{y}}_{i}\right)}^{2}},$$

(2) $$\mathrm{MAPE}=\frac{1}{m}\sum_{i=1}^{m}\left|\frac{y_{i}-{\hat{y}}_{i}}{y_{i}}\right|\times 100,$$

(3) $$R^{2}=1-\frac{\sum_{i=1}^{m}{\left(y_{i}-{\hat{y}}_{i}\right)}^{2}}{\sum_{i=1}^{m}{\left(y_{i}-{\bar{y}}_{i}\right)}^{2}}$$

where ‘m’ is the number of observations, ‘${\hat{y}}_{i}$’ is the predicted values, ‘$y_{i}$’ is the raw data of observations, and ‘${\bar{y}}_{i}$’ is the mean of ‘$y_{i}$’.

The FNN, SVR, GAM, and SLR algorithms were selected as the calibration methods in this study based on a pre-evaluation, and the specific results are shown in Table 3. The FNN, SVR, and GAM algorithms exhibited higher accuracies than the other algorithms. In contrast, correction-factor-based methods [35,39] showed the lowest accuracy and explanatory power. SLR performed poorly compared to FNN, SVR, and GAM but outperformed the correction-factor-based methods. Based on these results, four statistical methods were applied in the main experiment of this study, excluding physical-mechanism-based methods.

### 2.3. Combinations of Input Variables for Calibration

In addition to PM observations, this study considered RH, T, MA, and calibrated PM_2.5_ as input variables that affect PM concentrations. The reasons for selecting these variables are as follows: RH and T were selected because they have been identified as the most crucial variables for PM calibration in several previous studies. For PM LCS studies, the RH is considered an important variable in most calibrations. T was chosen because studies have shown that calibration performance improves when it is used in conjunction with RH [10]. In this study, we utilized RH and T observations provided by the Korea Meteorological Administration. These measurements were obtained from the meteorological observation station operated by the Korea Meteorological Administration located near the research site.

MA was selected as an input variable to account for seasonal variations in the PM calibration. The MA refers to the angle at which the sun reaches its zenith in the sky at a specific location. This has a significant influence on the intensity of sunlight and the length of the day, leading to climatic and seasonal changes. Therefore, MA can be considered a significant variable that can directly account for seasonal variations in the PM concentration in the calibration. It can continuously represent seasonal variations. Furthermore, time is based on local time zones, whereas MA is calculated using latitude and longitude information, thus directly relating to seasonal variations. This can lead to precise calibration performance.

Calibrated PM_2.5_ was selected as an additional input variable to calibrate PM_10_, allowing for an indirect consideration of PM_10_ measurements. As PM_2.5_ and PM_10_ define particles with sizes of 0–10 µm and 0–2.5 µm, respectively, PM_2.5_ is a fraction of PM_10_. This study utilized calibrated PM_2.5_ as an input variable to explain the concentration of PM_2.5_ and coarse PM_2.5-10_ (2.5–10 µm) separately. During the calibration process, the calibrated PM_2.5_ can indirectly account for changes in the concentration of PM_10_. In this case, the calibrated PM_2.5_, used for the PM_10_ calibration, was calibrated in the same manner as the PM_10_ calibration.

The combinations of input variables used for the calibration in this study are listed in Table 4. The results of each combination were compared and evaluated to determine their effectiveness. Comb-1 was calibrated by considering only RH, whereas Comb-2 also included T. Comb-3 considered MA in combination with RH and T, and Comb-4 included calibrated PM_2.5_ as a combination only for the calibration of PM_10_.

## 3. Results of PM LCS Calibration and Discussion

This study evaluated the accuracy of PM calibration using a combination of input variables and algorithms, including the FNN, SVR, GAM, and SLR. The four variable combinations used in the experiments consisted of RH, T, MA, and calibrated PM_2.5_ (Table 4).

### 3.1. Results of Calibration Based on Input Variable Combinations

PM_2.5_ calibration with Comb-3 performed the best across all sensors and algorithms. Comb-2 performed better than Comb-1 but performed worse than Comb-3. These results are listed in Table 5, which shows the average accuracy of all the algorithms for different combinations. For PM_2.5_ calibration, Comb-3 outperformed Comb-1 and Comb-2 by about 24% and 17% for MAPE, and by 4.4 and 1.9 µg/m^3^ for RMSE, respectively. Comb-3 also showed a higher R^2^ of about 0.14 and 0.05 compared to other combinations. Thus, it can be concluded that Comb-3 contributes to the development of a calibration model with high explanatory power and accuracy.

For PM_10_ calibration, Comb-3 and Comb-4 performed relatively better, with Comb-4 having the highest accuracy when including calibrated PM_2.5_. Table 6 shows the average accuracy of all the algorithms across different combinations for PM_10_ calibration. The PM_10_ calibration accuracies of all the algorithms followed the order of Comb-4, Comb-3, Comb-2, and Comb-1. The proposed Comb-4 showed 11.0, 6.5, and 1.3 µg/m^3^ lower RMSE and 23%, 12%, and 3% lower MAPE compared to Comb-1, Comb-2, and Comb-3, respectively. Comb-4 also showed an improvement in the explanatory power of the model, with a higher difference in R^2^ values of 0.16, 0.09, and 0.02 compared to Comb-1, -2, and -3. In comparison, the proposed Comb-3 was found to be more accurate than Comb-1 and Comb-2, regardless of the sensor type, but less accurate than Comb-4.

### 3.2. Results of Calibration Based on Algorithms

The algorithm with the best overall calibration performance across all sensor types and input variable combinations was the FNN. SVR and GAM performed similarly to FNN overall, with SVR outperforming GAM for PM_2.5_ and the opposite was true for PM_10_. In contrast, SLR showed the lowest overall accuracy and explanatory power compared to the other algorithms. Furthermore, all the algorithms improved their calibration performance with the proposed combinations of Comb-3 and Comb-4. This trend was consistently observed, regardless of the sensor and PM type.

**Figure 4:**
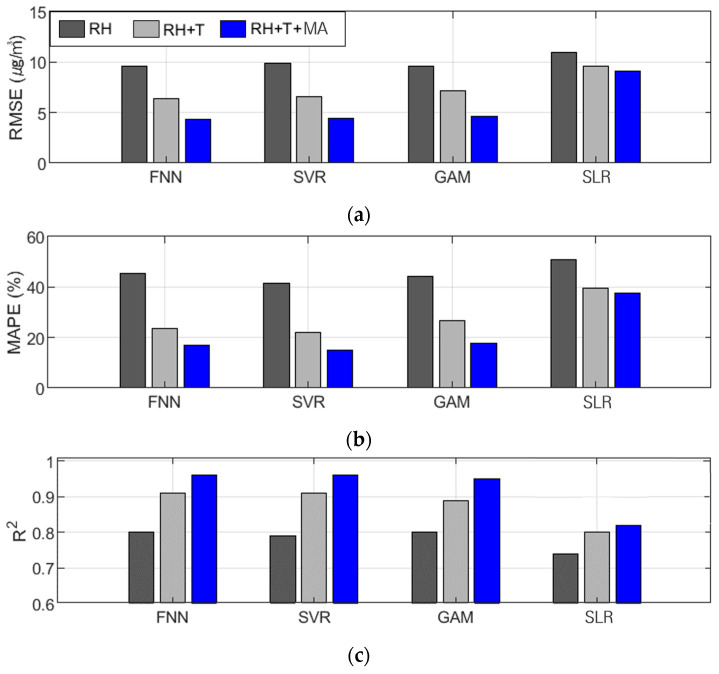
Tendency of performance variation in PM_2.5_ calibration based on input variable combinations of all algorithms: (**a**) RMSE (µg/m^3^), (**b**) MAPE (%), and (**c**) R^2^.

The PM_2.5_ calibration was followed by the FNN, SVR, GAM, and SLR (Table 7 and Figure 4). The FNN, SVR, and GAM performed similarly and the R² values of Comb-3 for these models were 0.96, 0.96, and 0.95, respectively, indicating high explanatory power. However, SLR showed a significant performance difference compared to the other algorithms, with RMSE, MAPE, and R² of 9.14 µg/m^3^, 37.53, and 0.82, respectively, for the proposed combination of variables.

**Table 7:** Performance of PM_2.5_ calibration based on algorithms.

Metric	Algorithm	Comb-1	Comb-2	Comb-3
RMSE (µg/m^3^)	FNN	9.60	6.42	4.29
	SVR	9.85	6.61	4.39
	GAM	9.57	7.19	4.58
	SLR	10.98	9.62	9.14
MAPE (%)	FNN	45.25	23.71	16.91
	SVR	41.43	21.94	15.05
	GAM	44.37	26.67	17.84
	SLR	50.96	39.45	37.53
R^2^	FNN	0.80	0.91	0.96
	SVR	0.79	0.91	0.96
	GAM	0.80	0.89	0.95
	SLR	0.74	0.80	0.82

**Figure 5:**
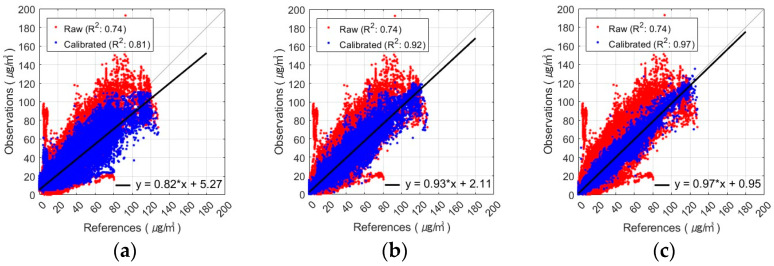
Scatterplot of FNN-based PM_2.5_ calibration with AM100 sensor: (**a**) Comb-1 as input variables, (**b**) Comb-2 as input variables, and (**c**) Comb-3 as input variables.

**Figure 6:**
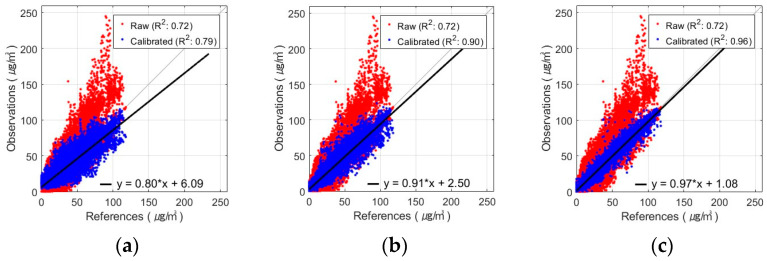
Scatterplot of FNN-based PM_2.5_ calibration with Sniffer 4D sensor: (**a**) Comb-1 as input variables, (**b**) Comb-2 as input variables, and (**c**) Comb-3 as input variables.

The best PM_2.5_ calibration results using the FNN are shown in Figure 5 and Figure 6. These figures show the results of the PM_2.5_ calibration based on the FNN using AM100 and Sniffer 4D sensors. These results confirm that the proposed Comb-3 can achieve more accurate calibration results than Comb-1 and Comb-2 for both sensors.

**Figure 7:**
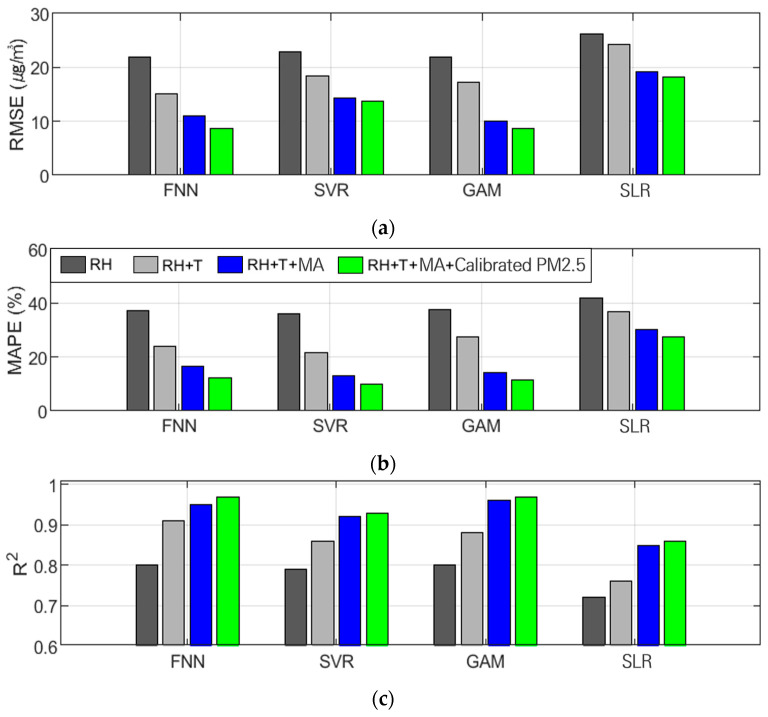
Tendency of performance variation in PM_10_ calibration based on input variable combinations of all algorithms: (**a**) RMSE (µg/m^3^), (**b**) MAPE (%), and (**c**) R^2^.

For PM_10_ calibration, the FNN and GAM algorithms generally yielded the most accurate calibration results, as shown in Table 8 and Figure 7. In calibrations using Comb-4, which showed the highest accuracy among all algorithms, the FNN and GAM achieved R² values of approximately 0.97 and 0.97. This performance was superior to that of SVR and SLR, which achieved R² values of 0.93 and 0.86. The GAM performed lower than the FNN in calibrations with Comb-1 and Comb-2 but outperformed the FNN in Comb-3 and -4, which are the proposed combinations. SVR performed better than SLR but worse than FNN and GAM, unlike PM_2.5_. Meanwhile, the SLR performed significantly worse than the other algorithms in terms of PM_10_ calibration.

**Table 8:** Performance of PM_10_ calibration based on algorithms.

Metric	Algorithm	Comb-1	Comb-2	Comb-3	Comb-4
RMSE (µg/m^3^)	FNN	21.91	15.11	11.01	8.68
	SVR	22.95	18.46	14.26	13.81
	GAM	22.01	17.16	10.06	8.58
	SLR	26.17	24.35	19.23	18.13
MAPE (%)	FNN	37.38	23.93	16.73	12.37
	SVR	36.20	21.56	13.08	10.00
	GAM	37.55	27.39	14.24	11.35
	SLR	41.90	36.74	30.11	27.66
R^2^	FNN	0.80	0.91	0.95	0.97
	SVR	0.79	0.86	0.92	0.93
	GAM	0.80	0.88	0.96	0.97
	SLR	0.72	0.76	0.85	0.86

**Figure 8:**
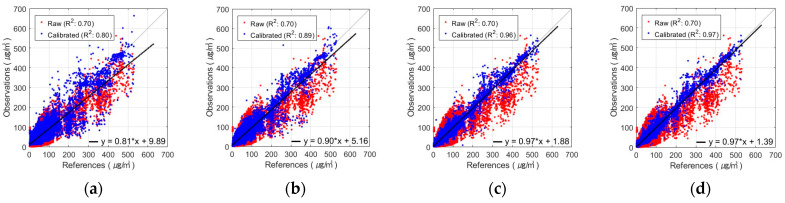
Scatterplot of FNN-based PM_10_ calibration with AM100 sensor: (**a**) Comb-1 as input variables, (**b**) Comb-2 as input variables, (**c**) Comb-3 as input variables, and (**d**) Comb-4 as input variables.

**Figure 9:**
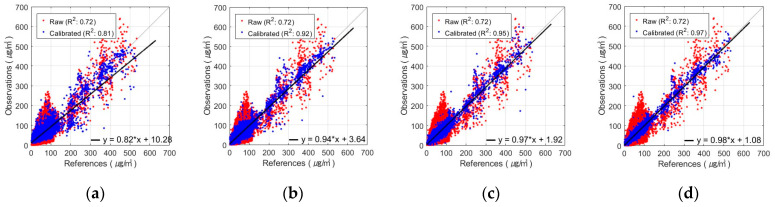
Scatterplot of FNN-based PM_10_ calibration with Sniffer 4D sensor: (**a**) Comb-1 as input variables, (**b**) Comb-2 as input variables, (**c**) Comb-3 as input variables, and (**d**) Comb-4 as input variables.

Figure 8 and Figure 9 show the results obtained using the FNN, one of the algorithms that performed the best in PM_10_ calibration for various combinations of input variables. These figures show the results of the AM100 and Sniffer 4D sensors. The results demonstrate that the calibration accuracy improved when the proposed variables, such as MA and calibrated PM_2.5_, were utilized (Comb-3 and Comb-4).

### 3.3. Results of Calibration Based on Seasonal Variation

This study investigated the effectiveness of the proposed input variable combinations for calibration by analyzing seasonal variations. The seasonal performance was analyzed in terms of the MAPE and R^2^ of the calibration using the FNN, which was the algorithm that exhibited the best performance for both PM_2.5_ and PM_10_. In other words, we assessed performance by analyzing the extent to which the MAPE and R^2^ of the corrected observations with FNN correction differed from those of the raw data. In this study, spring corresponded to March–May, summer to June–August, autumn to September–November, and winter to December–January. The climatic characteristics of each season in Korea where the observations were conducted are presented in Table 9.

**Figure 10:**
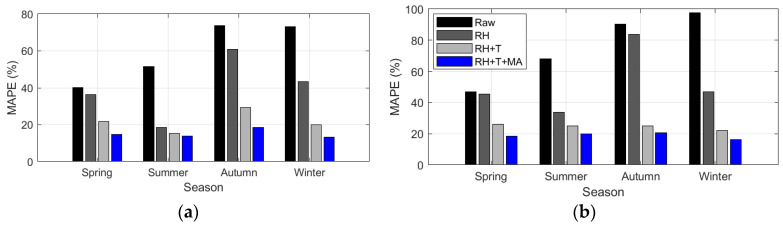
Seasonal variation of MAPE (%) based on input variable combinations in PM_2.5_ calibration: calibration using (**a**) AM100 sensor and (**b**) Sniffer 4D sensor.

For PM_2.5_, RH and T showed different contributions depending on the season, and the most precise calibration was achieved by applying MA, the variable proposed in this study, regardless of the season. In particular, the calibration using RH alone showed significant differences in accuracy depending on the season, as shown in Figure 10 and Table 10.

Comb-1 exhibited the best performance in summer, with an accuracy similar to the results obtained using T and MA. This can be attributed to the relatively low PM concentrations and very high RH in Korea during the summer, which are influenced by the rainy season. In winter, the accuracy of PM_2.5_ calibration was also significantly improved. However, unlike in summer, it was relatively low compared to the combination of T and MA. In contrast, there was only a slight improvement in spring and autumn when the weather was relatively dry, and the PM concentrations were high. In spring, autumn, and winter, Comb-2 showed a significant improvement in relative accuracy compared with Comb-1, indicating that summer is greatly influenced by RH. Particularly in spring and autumn, which have relatively high daily temperature ranges, the accuracy was lower than that in the other seasons. This can be explained by the fact that the maximum temperatures in spring and autumn are similar to those in summer, whereas the minimum temperature is similar to that in winter. This makes it difficult for temperatures to account for seasonal characteristics completely. Comb-3, on the other hand, was stable regardless of the season and had the highest relative accuracy. Comb-3 improved the MAPE accuracy by 63–82% (AM100) and 58–75% (Sniffer 4D) over the raw data (Table 10).

**Figure 11:**
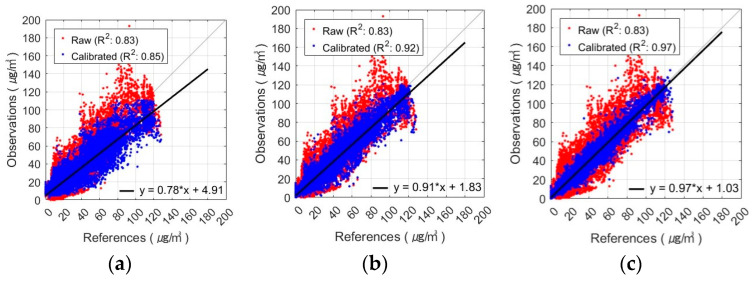

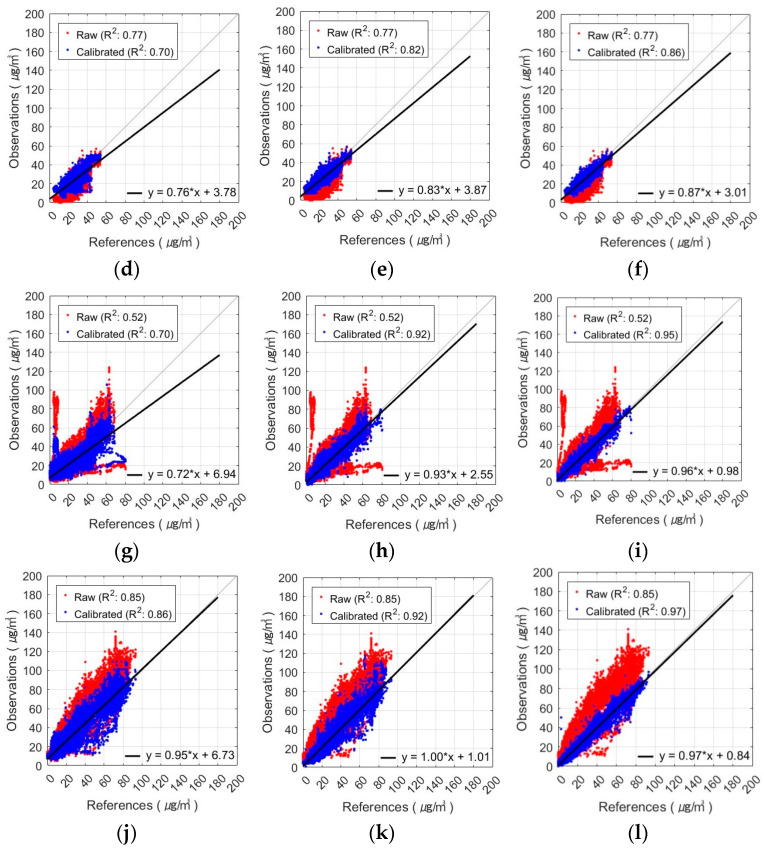
Seasonal variation of FNN-based AM100-PM_2.5_ calibration results with different input variable combinations: (**a**–**c**) spring result by combination, (**d**–**f**) summer result by combination, (**g**–**i**) autumn result by combination, and (**j**–**l**) winter result by combination (from left to right, these are Comb-1, -2, and -3).

The seasonal R-squared for the AM100-PM_2.5_ observations with the FNN-based calibration model can be seen in Figure 11. Comb-1 showed an improvement in R^2^ compared to the raw data in spring, autumn, and winter, especially in autumn. In contrast, in summer, the R^2^ value decreased from 0.77 to 0.70. This indicates that the FNN-calibrated model, considering only the RH variable, exhibited reduced explanatory power for the summer observations. In Comb-2, temperature was also taken into account, which enhanced the explanatory power in all seasons compared to Comb-1. The Comb-3 exhibited the highest R^2^ values across all seasons and the most explanations of PM_2.5_ concentrations. Spring, autumn, and winter especially showed high values of R^2^ in the range of 0.95-0.97, with autumn improving by 0.43 over the raw data. Observations in summer had lower explanatory power than the other seasons for all input variable combinations but showed improved performance with the addition of T and MA compared to raw data.

**Figure 12:**
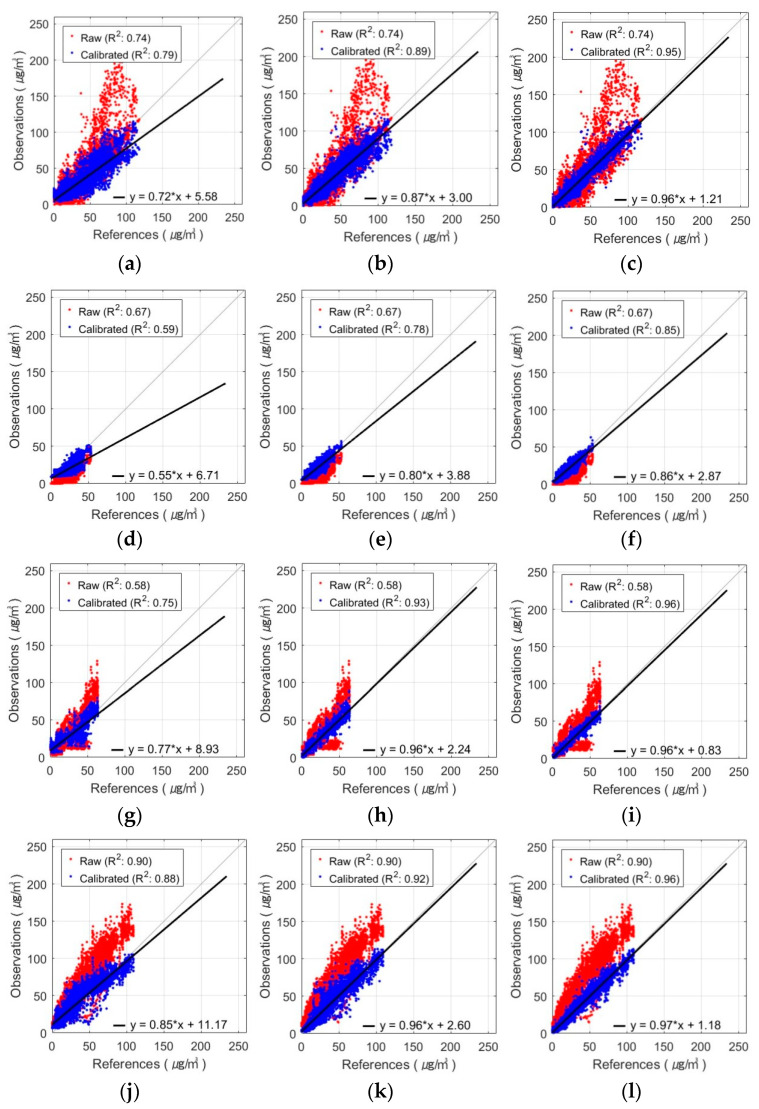
Seasonal variation of FNN-based Sniffer4D-PM_2.5_ calibration results with different input variable combinations: (**a**–**c**) spring result by combination, (**d**–**f**) summer result by combination, (**g**–**i**) autumn result by combination, and (**j**–**l**) winter result by combination (from left to right, these are Comb-1, -2, and -3).

The seasonal R-squared of the Sniffer4D-PM_2.5_ observations with the FNN-based calibration model is shown in Figure 12. Comb-1 exhibited improved explanatory power over the raw data in spring and autumn but decreased from 0.67 to 0.59 and from 0.90 to 0.88 in summer and winter, respectively. Compared to the AM100 sensor, the Sniffer4D sensor showed reduced explanatory power for both winter and summer observations. Comb-2 showed an improvement in the value of R^2^ compared to Comb-1. However, there was a seasonal difference, with higher values in fall and winter and lower values in spring and summer. Comb-3 decreased the difference in R^2^ between spring, autumn, and winter to 0–0.01. In all seasons, the combination of RH, T, and MA variables explained PM_2.5_ concentrations the most.

**Figure 13:**
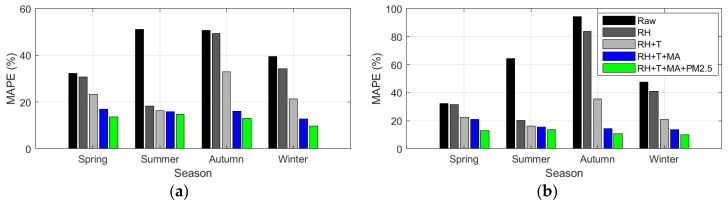
Seasonal variation of MAPE (%) based on input variable combinations in PM_10_ calibration: calibration using (**a**) AM100 sensor and (**b**) Sniffer 4D sensor.

The relationship of each variable to the seasonal PM_10_ calibration results showed similar patterns to PM_2.5_; that is, RH and T showed varying performances across different seasons. In contrast, by considering the proposed variable MA and calibrated PM_2.5_, more accurate calibration results were achieved regardless of the season (Figure 13 and Table 11).

The PM_10_ calibration based on seasonal input variables revealed the following trends: Comb-1 shows a significant decrease in MAPE in summer but a relatively smaller decrease in MAPE in other seasons. Comb-2, which used RH and T, demonstrated a consistently higher accuracy across all seasons than Comb-1. However, Comb-2 still showed higher errors in all seasons, except for summer, with the highest error levels in autumn. Comb-3 and Comb-4 exhibited consistently high accuracies across all seasons. Comb-4 demonstrated MAPE accuracy enhancements of 60–89% points (AM100) and 58–75% points (Sniffer 4D) compared to the raw observations (Table 11).

**Figure 14:**
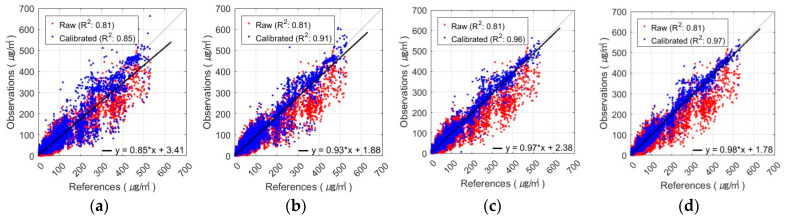

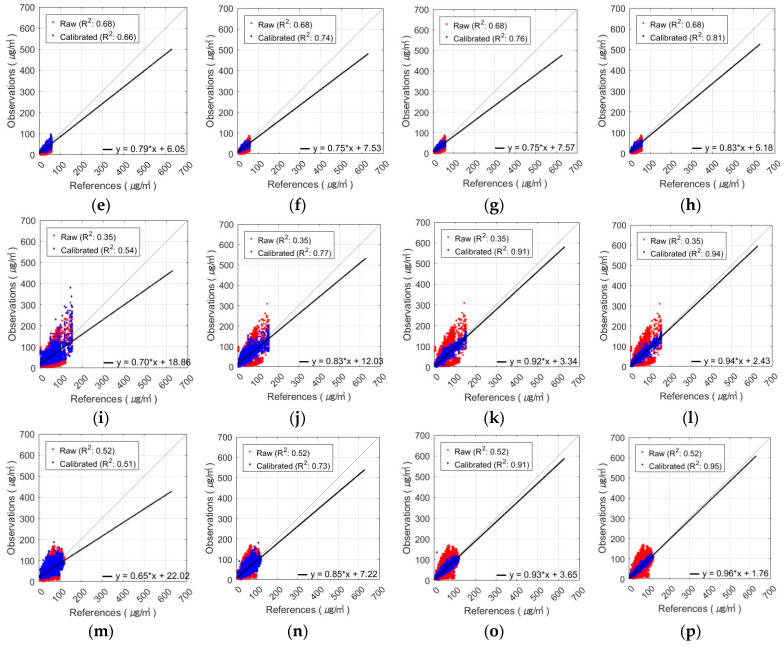
Seasonal variation of FNN-based AM100-PM_10_ calibration results using different input variable combinations: (**a**–**d**) spring result by combination, (**e**–**h**) summer result by combination, (**i**–**l**) autumn result by combination, and (**m**–**p**) winter result by combination (from left to right, these are Comb-1, -2, -3, and -4).

The calibration of AM100-PM_10_ observations with the FNN-based calibration model resulted in the following seasonal R-squared values (Figure 14). In Comb-1, the value of R^2^ increased in spring and autumn but decreased slightly in summer and winter. Similar to PM_2.5_, PM_10_ concentrations also showed variations in explanatory power across seasons. Comb-2 showed a higher R^2^ value of 0.91 in spring but lower values in the other seasons, ranging from 0.73 to 0.77. In Comb-3, the value of R^2^ improved in all seasons, especially in autumn and winter, increasing from 0.77 to 0.91 and 0.73 to 0.91, respectively. Comb-4 exhibited the highest R^2^ values in all seasons and further enhanced the explanatory power of PM_10_ by incorporating calibrated PM_2.5_.

**Figure 15:**
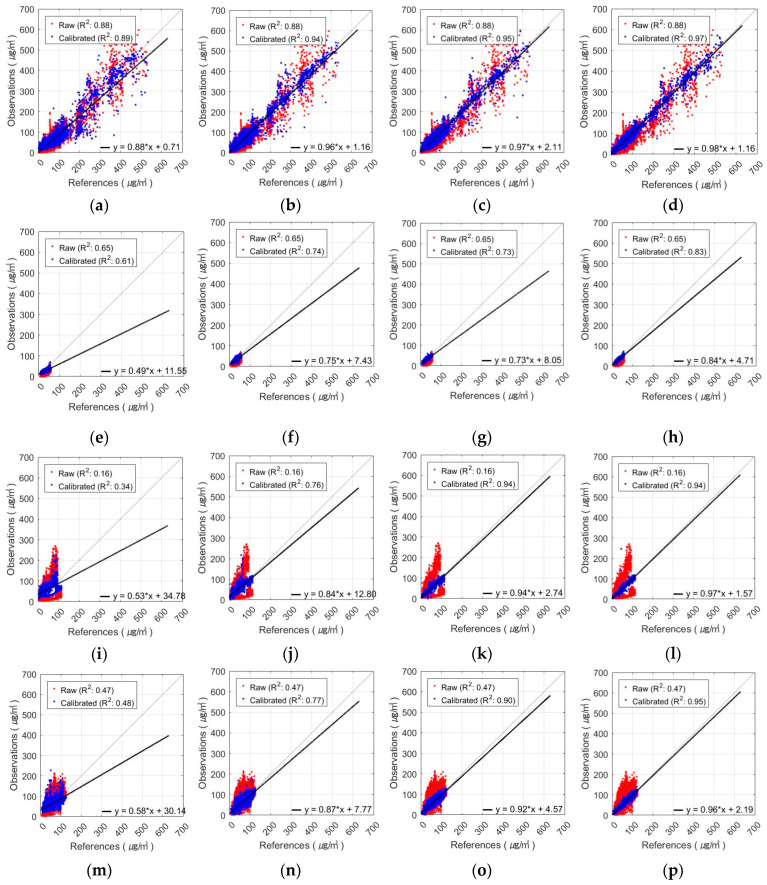
Seasonal variation of FNN-based Sniffer4D-PM_10_ calibration results using different input variable combinations: (**a**–**d**) spring result by combination, (**e**–**h**) summer result by combination, (**i**–**l**) autumn result by combination, and (**m**–**p**) winter result by combination (from left to right, these are Comb-1, -2, -3, and -4).

The seasonal results of calibrating the Sniffer4D-PM_10_ observations with the FNN-based calibration model are shown in Figure 15. Comb-1 showed an improvement in the value of R^2^ in spring, autumn, and winter, but decreased slightly in summer. In Comb-2, the explanatory power improved in all seasons compared to Comb-1. Moreover, similar to the AM100 sensor, the spring observations showed higher explanatory power than the other seasons. Comb-3 showed better calibration performance in all seasons, especially in autumn and winter. The value of R^2^ decreased slightly in summer compared to Comb-2 by 0.01 but improved by 0.8 over the raw data. Comb-4 showed the highest R^2^ values in all seasons, indicating that it was the best combination of input variables to describe PM_10_.

## 4. Conclusions

This study proposed a methodology to enhance the calibration performance of the PM LCS. The proposed methodology considers the MA and calibrated PM_2.5_ as novel input variables in a statistical calibration model. The MA was selected as a factor to consider the seasonal variation in the PM concentration and calibrated PM_2.5_ was selected to account for the concentration of PM_2.5_ and coarse PM_2.5_ in PM_10_ calibration. To assess the effectiveness of the proposed input variables, four combinations of input variables were applied to the FNN, SVR, GAM, and SLR algorithms.

The results showed that in PM calibration, the variable combination including MA outperformed the combination without MA. For PM_2.5_, the combination that considered RH, T, and MA had the highest relative accuracy and explanatory power of all algorithms. For PM_10_ calibration, the combination that considered the MA outperformed the combination that only considered RH and T. The highest performance in PM_10_ calibration was observed when calibrated PM_2.5_ was included, specifically when RH, T, MA, and calibrated PM_2.5_ were utilized. These results suggest that MA can explain the variation in the PM concentration over the seasons and can be effectively considered in the calibration. In addition, the PM_10_ calibration model using calibrated PM_2.5_ variables can efficiently account for the characteristics of a subset of PM_10_.

Among the calibration models used, the FNN performed the best across all PM types, sensors, and variable combinations, with R² values of 0.96 and 0.97 for PM_2.5_ and PM_10_ calibrations, respectively, indicating high explanatory power. SVR and GAM performed similarly to FNN, with GAM showing a slightly poorer performance for PM_2.5_ and SVR performing slightly poorly for PM_10_. In contrast, SLR, the regression model, performed worse overall than the machine learning models.

The proposed methodology is expected to help efficiently calibrate the PM LCS in different seasons and environments. In this study, we used one year of data to account for seasonal variations. In the future, we plan to collect data for less than a year and develop an in situ calibration methodology to ensure accuracy. We will also explore methods for independently assessing PM_2.5_ and PM_10_.

## Figures and Tables

**Table 1:** Specification of PM LCS (AM100 and Sniffer 4D).

Specification	Air-Ruler AM100	Sniffer 4D
Detection method	light scattering	light scattering
Range	0.3–1000 µg/m^3^	0.3–1000 µg/m^3^
Minimum detection limit	1 µg/m^3^	1 µg/m^3^
Measurement resolution	0.1 µg/m^3^	1 µg/m^3^
Time resolution (observation)	1 min	1 s
Time resolution (experiment)	1 min	1 min (mean)
Additional observations	temperature, humidity	temperature, humidity
Observation period	January 2021 to December 2022	January 2021 to December 2022 (3 days per week)

**Table 2:** Statistics of PM_2.5_ and PM_10_ concentration collected from sensors (January 2021–December 2022; Unit: µg/m^3^).

Sensors	Target	Num of Observations Used	Mean	* SD	* SE	Proportion of Observations Below * MDL
BAM-1020 (Reference)	PM_2.5_	11,737	28.2193	20.8432	0.1924	2.71%
	PM_10_	11,697	53.0394	47.9211	0.4431	0.23%
Air-ruler AM100	PM_2.5_	11,737	28.22	20.84	0.19	2.71%
	PM_10_	11,697	53.04	47.92	0.44	0.23%
Sniffer 4D	PM_2.5_	4901	29.66	21.98	0.31	3.16%
	PM_10_	4901	55.16	50.90	0.72	0.31%

* SD stands for Standard Deviation, SE stands for Standard Error, and MDL stands for Minimum Detection Limit.

**Table 3:** Results of pre-evaluation using RH and selection of calibration method.

Algorithm	Target	Sensor	RMSE (µg/m^3^)	MAPE (%)	R^2^	Selection
FNN [25]	PM_2.5_	AM100	9.04	42.21	0.81	Accepted
		Sniffer 4D	10.17	48.29	0.79	
		Mean	9.61	45.25	0.80	
	PM_10_	AM100	21.24	34.91	0.80	
		Sniffer 4D	22.57	39.86	0.81	
		Mean	21.91	37.39	0.81	
SVR [43]	PM_2.5_	AM100	9.34	39.69	0.80	Accepted
		Sniffer 4D	10.37	43.18	0.78	
		Mean	9.86	41.44	0.79	
	PM_10_	AM100	22.05	33.02	0.79	
		Sniffer 4D	23.85	39.38	0.78	
		Mean	22.95	36.20	0.79	
GAM [26]	PM_2.5_	AM100	9.01	42.61	0.82	Accepted
		Sniffer 4D	10.12	46.14	0.79	
		Mean	9.57	44.38	0.81	
	PM_10_	AM100	21.23	35.11	0.80	
		Sniffer 4D	22.79	39.99	0.80	
		Mean	22.01	37.55	0.80	
SLR [40]	PM_2.5_	AM100	10.45	47.11	0.75	Accepted
		Sniffer 4D	11.50	54.80	0.73	
		Mean	10.98	50.96	0.74	
	PM_10_	AM100	25.41	40.92	0.71	
		Sniffer 4D	26.92	42.88	0.72	
		Mean	26.17	41.90	0.72	
Correction factor 1 [31]	PM_2.5_	AM100	12.81	50.05	0.67	Rejected
		Sniffer 4D	15.55	61.82	0.69	
		Mean	14.18	55.94	0.68	
	PM_10_	AM100	27.26	40.47	0.67	
		Sniffer 4D	29.69	47.81	0.72	
		Mean	28.48	44.14	0.70	
Correction factor 2 [36]	PM_2.5_	AM100	14.25	56.09	0.61	Rejected
		Sniffer 4D	17.13	65.87	0.63	
		Mean	15.69	60.98	0.62	
	PM_10_	AM100	31.09	43.55	0.61	
		Sniffer 4D	32.01	50.09	0.67	
		Mean	31.55	46.82	0.64	

**Table 4:** Combinations of input variables consisting of RH, T, MA, and calibrated PM_2.5._

Combination No.	Calibration Target	Variables
Comb-1	PM_2.5_, PM_10_	RH
Comb-2	PM_2.5_, PM_10_	RH + T
Comb-3	PM_2.5_, PM_10_	RH + T + MA
Comb-4	PM_10_	RH + T + MA + Calibrated PM_2.5_

**Table 5:** Performance of PM_2.5_ calibration based on input variable combinations.

Combination	Sensor	RMSE (µg/m^3^)	MAPE (%)	R^2^
Raw data	AM100	13.97	57.86	0.72
	Sniffer 4D	29.05	71.85	0.71
	Mean	21.51	64.86	0.72
Comb-1 (RH)	AM100	10.54	48.10	0.77
	Sniffer 4D	9.46	42.90	0.80
	Mean	10.01	45.51	0.79
Comb-2 (RH + T)	AM100	7.06	26.57	0.88
	Sniffer 4D	7.85	29.31	0.87
	Mean	7.46	27.94	0.88
Comb-3 (RH + T + MA)	AM100	5.37	20.98	0.93
	Sniffer 4D	5.82	22.69	0.92
	Mean	5.60	21.84	0.93

**Table 6:** Performance of PM_10_ calibration based on input variable combinations.

Combination	Sensor	RMSE (µg/m^3^)	MAPE (%)	R^2^
Raw data	AM100	28.29	42.81	0.67
	Sniffer 4D	31.38	50.54	0.71
	Mean	29.84	46.68	0.69
Comb-1 (RH)	AM100	22.49	35.99	0.77
	Sniffer 4D	24.03	40.53	0.78
	Mean	23.26	38.26	0.78
Comb-2 (RH + T)	AM100	18.70	27.88	0.84
	Sniffer 4D	18.84	26.93	0.86
	Mean	18.77	27.41	0.85
Comb-3 (RH + T + MA)	AM100	13.49	18.71	0.91
	Sniffer 4D	13.79	18.37	0.92
	Mean	13.64	18.55	0.92
Comb-4 (RH + T + MA + Calibrated PM_2.5_)	AM100	12.31	16.22	0.93
	Sniffer 4D	12.29	14.47	0.94
	Mean	12.30	15.35	0.94

**Table 9:** Meteorological characteristics of seasons in South Korea.

Season (Month)	Spring (Mar.–May)	Summer (Jun.–Aug.)	Autumn (Sep.–Nov.)	Winter (Dec.–Feb.)
Humidity (average RH)	Dry (59.0%)	Humid (75.2%)	Dry (65%)	Dry (59.8%)
Temperature (average)	13.2	24.5	14.8	0.2
Daily temperature range	10.4	6.7	9.4	8.8

**Table 10:** Enhancement rate of MAPE (%) over raw data of each season according to input variable combinations in PM_2.5_ calibration.

Combination	Enhancement Rate of MAPE (%)							
	AM100 Sensor				Sniffer 4D Sensor			
	Spring	Summer	Autumn	Winter	Spring	Summer	Autumn	Winter
Comb-1	9	64	17	41	3	50	7	52
Comb-2	46	70	60	73	44	63	72	77
Comb-3	63	73	75	82	61	70	77	83

**Table 11:** Enhancement rate of MAPE (%) over raw data of each season according to input variable combinations in PM_10_ calibration.

Combination	Enhancement Rate of MAPE (%)							
	AM100 Sensor				Sniffer 4D Sensor			
	Spring	Summer	Autumn	Winter	Spring	Summer	Autumn	Winter
Comb-1	2	69	11	14	5	64	3	13
Comb-2	30	75	62	56	62	68	35	46
Comb-3	34	76	85	71	47	69	68	68
Comb-4	60	79	89	79	58	71	74	75

## Data Availability

The original meteorological data presented for calibration in the study are openly available on the Korea Meteorological Administration website at https://data.kma.go.kr/cmmn/main.do (accessed on 9 May 2024).
